# Consensus derived client outcomes and clinician actions for youth online chat mental health services: a Delphi study

**DOI:** 10.3389/fdgth.2025.1671364

**Published:** 2025-12-16

**Authors:** Sonia Curll, Kelly Mazzer, Sabina Albrecht, Skye Barbic, Amanda Fitzgerald, Kairi Kõlves, Nic Telford, Nickolai Titov, Debra Rickwood

**Affiliations:** 1 Faculty of Health, University of Canberra, Canberra, ACT, Australia; 2 headspace National Youth Mental Health Foundation, Melbourne, VIC, Australia; 3 Faculty of Medicine, The University of British Columbia, Vancouver, BC, Canada; 4 Foundry, Vancouver, BC, Canada; 5 School of Psychology, University College Dublin, Dublin, Ireland; 6 Australian Institute for Suicide Research and Prevention, School of Applied Psychology, Griffith University, Brisbane, QLD, Australia; 7 Faculty of Medicine, Health, and Human Sciences, Macquarie University, Sydney, NSW, Australia

**Keywords:** online chat, youth mental health, outcome measurement, Delphi, digital mental health, fidelity, evaluation

## Abstract

**Introduction:**

Online chat services have increased mental health care access for young people (12–25 years), yet their effectiveness remains unclear. This is partly due to a lack of consensus about primary client outcomes and clinician actions facilitating positive service outcomes. This study sought to identify (a) outcomes most important for young people accessing mental health support via online chat, and (b) clinician actions most relevant to achieving these outcomes.

**Method:**

A comprehensive list of potential outcomes and actions was developed through literature review and consultation with youth online chat service providers. A three-round Delphi study was conducted with three panels of youth, researchers, and clinicians (*n* = 100; 84% retention rate), primarily from Australia and Ireland. Consensus was reached if ≥75% of participants within at least two panels rated an outcome/action as very important or essential.

**Results:**

Eleven client outcomes reached consensus: *Feeling heard and validated; Reduced distress; Increased help-seeker capacity; Feeling safe; Optimism and hope; Connection with clinician and service; Feeling better; Reduced hopelessness; Reduced overwhelm; Increased coping;* and *Goals, answers and direction*. Fifteen clinician actions reached consensus: *Manage risk; Respect diversity; Validation; Welcoming environment; Active listening; Manage distress; Compassion; Checking in; Give choice; Youth friendly; Set expectations and focus; Provide resources; Holistic approach; Highlight strengths;* and *Problem-solving.*

**Conclusions:**

The identified client outcomes and clinician actions offer preliminary guidance for monitoring and evaluating youth online chat support. Future research should test and refine these domains within service contexts to inform robust measurement tool development for evaluating youth online chat services.

## 1 Introduction

Poor mental health for young people is a major risk factor for physical and mental health issues later in life (1, 2), with approximately two-thirds of mental disorders starting before the age of 25 years (3). Early intervention can prevent progression of mental health conditions and enhance long-term health outcomes (4, 5). However, a complex array of individual, social, and structural barriers to service access means that rates of mental health service use are disproportionately low among young people (6). This highlights an urgent need for effective and easily accessible interventions to support youth mental health (7).

Digital mental health interventions are well positioned to address common barriers to help-seeking and enable early access to support (8, 9). Young people often view them as safer, more private, less stigmatising, and more empowering than traditional face-to-face services (10, 11). As an accessible first point of contact, digital services can provide timely support for mental health concerns, reducing the likelihood of escalation and easing pressure on higher-intensity, in-person interventions (9, 12, 13). Digital services can thus contribute to more efficient and sustainable mental health care for young people (14, 15).

One type of digital mental health service gaining popularity among young people is online chat support (16–18). Primary care models for youth mental health care, such as headspace in Australia, are diversifying and offering alternate modes of support, including online chat (17–19). The growing popularity of online chat is reflected in usage data: for example, headspace's digital service, eheadspace, recorded almost 40,000 occasions of service in 2024, the majority through online chat (20). Similar patterns are observed internationally, with increasing uptake of online chat support among young people from services such as Jigsaw Ireland (21), Foundry's virtual service in Canada (18), and Kooth in the UK (22). While the format and delivery of these services varies, this paper focuses on online chat support delivered synchronously (in real-time) by trained mental health clinicians via instant messaging on secure, web-based platforms (16, 23).

The distinctive characteristics of online chat support create both opportunities and challenges for mental health care. Young people accessing online chat tend to report higher levels of distress and disclosure compared with in-person services (24–26). This may be due to factors associated with online chat, including the immediacy of access, feelings of reduced emotional proximity and increased privacy, and greater sense of autonomy and control (27–29). However, the same features that enhance accessibility can also present clinical challenges. The lack of non-verbal cues, slower communication pace, and single-session format may limit the depth of assessment and intervention (16). Technical issues and other distractions can further disrupt the therapeutic process (28), requiring clinicians to respond flexibly within a constrained timeframe. These complexities highlight the need for adapted processes and specialised clinical skills for providing online chat support (23, 30).

The effectiveness of online chat services for young people remains largely unknown, primarily due to inadequate measurement approaches not tailored for this unique context (8, 10, 31). A fundamental challenge lies in determining what outcomes best reflect meaningful change for young people in the online chat environment (30). This uncertainty is compounded by practical and ethical issues, particularly as traditional evaluation methods involving self-report pre/post surveys are difficult to implement within single-session online chat services and can create barriers to both user engagement and the provision of timely support (32).

Several studies have begun to address these measurement challenges through innovative approaches across different service contexts. The Session Wants and Needs Outcome Measure (SWAN-OM) was developed for young people using a data driven approach within a specific single-session digital mental health service (Kooth in the UK) (33, 34). While this represents an important advance, it did not incorporate clinician input and may have limited generalisability beyond that context. Building on this work, the Adult SWAN-OM was developed for single-session digital mental health interventions for adults using large language models and co-design methods, successfully integrating perspectives from both service users and clinicians (35). However, the priorities and needs of young people may differ from those of adults. Furthermore, given the practical challenges of implementing self-report tools in online chat services, it will be important for future research to explore emerging techniques that offer potential for automated evaluation, such as machine learning [e.g., (36, 37)].

Another major measurement gap is the absence of ways to assess clinicians' therapeutic processes and behaviours (clinician actions) during online chat sessions (8, 31). Process evaluation is a vital component of service evaluation to determine how well an intervention works and how it can be improved (38). Assessing clinician actions represents a key focus within process evaluation, as it helps identify the therapeutic mechanisms that contribute to positive outcomes (38). While there is an assumption that typical (in-person) therapeutic processes will transfer to and appropriately apply within the online environment, this has not been demonstrated, and the distinct characteristics of online chat (e.g., usually single session, anonymous) suggest different processes may be involved (23). Traditional process measures are poorly suited to the online chat context because they are often intrusive, rely on self-report, lack real-time assessment capability, and are typically designed for specific manualised treatments rather than the diverse and transdiagnostic approaches typically used in online chat settings (31). Moreover, most existing measures have been developed for adult populations, with limited validation or adaptation for youth-specific contexts.

Robust outcome and process measures tailored to the youth online chat setting are urgently needed. Identifying key client outcomes and clinician actions for young people accessing online chat services represents a vital first step toward this goal. Current measurement approaches have focused primarily on predetermined outcomes without sufficient input from young people, while clinician action measurement remains largely unexplored in this context. In addition to meeting growing accountability demands, robust measurement approaches will inform the development of evidence-based models of practice and ensure services remain responsive to the diverse and evolving needs of young people.

This study aimed to develop international consensus on priority client outcomes and clinician actions for youth online chat services, integrating perspectives from young people, researchers, and clinicians. Specifically, we sought to identify (a) the most important *client outcomes* for young people accessing mental health support via online chat (i.e., what do young people hope to gain from accessing online chat?), and (b) the most important *clinician actions* in chat interactions to achieve these outcomes (i.e., what should clinicians do to contribute to positive outcomes?). We used the Delphi method because it provides an evidence-based and systematic process for integrating diverse interest-holder perspectives into group consensus (39). The anonymous, online format enabled us to integrate distinct perspectives from geographically dispersed young people, clinicians, and researchers, while addressing potential power imbalances that could undermine the consensus process (40). By establishing consensus across key interest-holder groups, this study provides essential groundwork for researchers and service providers to develop meaningful measurement tools that can improve service accountability and effectiveness.

## 2 Materials and methods

### 2.1 Study design

We conducted a Delphi study with three panels meeting the following inclusion criteria: 1) young people (aged 15–25 years) using or seeking digital mental health support, 2) researchers with expertise in youth mental health, digital service provision, or outcome measurement, and 3) clinicians with experience delivering digital mental health support to young people. We surveyed each panel separately to explore similarities and differences in perspectives across panels, which is crucial for designing outcome measurement approaches that are meaningful to all interest-holders (41).

The University of Canberra's Human Research Ethics Committee provided ethical approval (Project ID: 14186). The study was not prospectively registered. Reporting follows the ACcurate COnsensus Reporting Document (ACCORD) guidelines (42; see completed checklist in Supplementary Materials). The project team was led by DR and comprised researchers with experience in consensus methodology (SC, KM, DR, KK) and experts in youth mental health, digital mental health services, outcome evaluation, and user engagement (KM, SA, SB, AF, KK, NTe, Nti, DR).

This study was conducted in partnership with three youth mental health services: eheadspace (Australia), Jigsaw (Ireland), and Foundry (Canada). All three operate within primary care youth mental health models that provide free, accessible, early intervention support to young people aged 12–25 years, offering online chat support alongside other modalities (e.g., email, phone) and community-based services (18, 19, 21).

### 2.2 Panel recruitment

The core research team (SC, KM, DR) coordinated panelist recruitment between September and October 2024, with a target of 20–40 participants for each panel, following recommendations for Delphi studies (39).

#### 2.2.1 Youth panel

eheadspace (Australia) and Jigsaw (Ireland) recruited youth panelists via email invitations to service users aged 15–25 years who had consented to be contacted for research opportunities in 2024. We focused on this age range based on ethical considerations regarding independent consent, and to recruit participants who could provide detailed insights based on their service experience (experts-by-experience). We offered youth panelists a small payment (equivalent AU$15 per round) to acknowledge their valuable contribution as experts-by-experience and to align with research participation guidelines (43).

#### 2.2.2 Clinician panel

eheadspace and Jigsaw recruited clinician panelists via staff communication channels and offered them time to participate during paid work hours.

#### 2.2.3 Researcher panel

The research team identified potential researcher panelists through project investigator recommendations and literature searches of relevant peer-reviewed studies published since 2020, reflecting the significant increase in online chat service usage during and after the COVID-19 pandemic. We sent email invitations to identified researchers. No financial incentives were offered, as participation in research is anticipated in academic positions.

All potential panelists registered via Qualtrics and received a plain language statement outlining the research aims, participation guidelines, and voluntary anonymous participation. Eligibility was verified by the research team based on the inclusion criteria outlined above. All eligible individuals who expressed interest were invited to participate and became panelists upon completing the first survey round.

### 2.3 Survey development

We developed comprehensive item pools for (a) client outcomes and (b) clinician actions based on literature findings and research team expertise (44). We conducted a targeted scoping review to identify existing core outcome sets, outcome measures, and service program theory frameworks relevant to youth single-session online chat support. We focused on sources developed since 2020, as youth online chat services have evolved rapidly in response to increased demand for digital mental health support during and after the COVID-19 pandemic. The scoping review identified the following key sources: a core outcome set developed for crisis helplines (45); the SWAN-OM for children and young people, a recently validated single-session outcome measure developed for Kooth (33, 34); a systematic review of the youth online chat counselling literature (31); the eheadspace evaluation framework (46); the Jigsaw live chat programme theory model (47), and the theory of change for Kooth (22). We retained items that were: a) relevant to any young person (e.g., not demographic-specific) and b) relevant to any youth online chat service (e.g., not context-specific). The core research team consolidated the items and developed labels and descriptions for the survey. The full research team, including representatives from headspace and Jigsaw, then reviewed and refined the survey to ensure completeness and appropriate language for this service context. Survey materials were not piloted. The full list of items included in the survey, mapped to their sources, is presented in Supplementary Tables S1, S2.

### 2.4 Survey Administration

We conducted three online survey rounds between October and December 2024 using Welphi (https://www.welphi.com), a secure platform developed for Delphi method surveys. Unique survey links were distributed via email, and each survey remained open for two weeks, with two weeks between rounds for analysis. Participants provided informed consent at the start of each survey. Participants participated individually and remained anonymous to each other throughout the Delphi process. For monitoring and feedback purposes, the core research team had access to participant identities, but individual responses were kept anonymous. No items were removed during the consensus process. Minor modifications to item labels and descriptions were made between rounds based on participant feedback (see Supplementary Table S3).

#### 2.4.1 Round 1

We asked participants to rate the importance of 26 outcomes for young people accessing digital mental health support via online chat on a 5-point scale (1 = not important or not realistic, 2 = less important, 3 = important, 4 = very important, 5 = essential). In the survey instructions, we defined *online chat* as 1-on-1 live (real-time) support chatting with a trained clinician through instant messages (web-chat), and *client outcomes* as the wants and needs of young people accessing mental health support via online chat. We provided free-text comment boxes for each outcome to enable participants to explain their responses. After rating the outcomes, participants had the opportunity to propose new outcomes and provide general feedback. We also collected demographic data (gender, age, country of residence), and experience data: (researcher panel) years of experience in youth mental health, digital mental health services, and/or outcome measurement research; (clinician panel) years of experience providing digital mental health services, experience with online chat support; (youth panel) frequency, recency, and type of support received from digital mental health services.

#### 2.4.2 Round 2

We presented participants with a summary of the Round 1 results, including endorsed items from each panel and key themes from open-text comments. Before re-rating each item, participants viewed their own rating alongside their panel's aggregate percentages and anonymous open-text comments. We introduced two additional client outcomes identified from Round 1 qualitative feedback (see Supplementary Table S3). Participants were then asked to rate the importance of 21 clinician actions in online chat with young people accessing digital mental health support using the same 5-point scale, with free-text comment options. *Clinician actions* were defined as the processes or behaviours in online chat that contribute to young people's desired outcomes.

#### 2.4.3 Round 3

Following the Round 2 approach, we presented participants with a summary of the Round 2 results, including detailed feedback from their panel, before asking them to re-rate each clinician action. We introduced two additional clinician actions identified from Round 2 qualitative feedback (see Supplementary Table S3).

### 2.5 Statistical analysis

We performed statistical analysis using SPSS version 30. We analysed data separately for each panel, then aggregated and pooled results to ensure equal representation regardless of panel size. We calculated descriptive statistics (aggregate percentages, means, and standard deviations) for each panel's responses after each round. We determined consensus *a priori* as ≥75% of participants from at least two panels rating an item as very important or essential. While there is no gold standard definition of consensus (48), thresholds between 70%–80% are recommended (44, 49, 50), and this definition has been successfully applied in Delphi studies with similar panel composition and process design (45, 51).

We classified items as conflicts when they reached consensus in at least one panel but showed ≥25 percentage point differences between any two panels in the proportion rating as very important or essential (45, 51). The purpose of this classification process was to identify potentially important between-panel differences for further consideration, consistent with recommendations for multi-panel Delphi processes (52). This classification did not affect the consensus process, and items could meet both consensus and conflict criteria.

We analysed qualitative data from open-text responses using inductive qualitative content analysis. Responses were coded into themes in an iterative process using Excel by the first author [SC] and reviewed by the second author [KM]. We used these findings to refine items, introduce new items, and explore potential reasons for conflicts. We also incorporated summaries of open-text responses into participant feedback.

Post hoc grouping of consensus items into thematic domains was conducted through content mapping by two authors [SC, KM] and refined through team discussion to aid interpretation (52). This categorisation was not part of the Delphi process and did not influence participant ratings.

## 3 Results

### 3.1 Panel members

One hundred participants completed Round 1: 49 young people, 16 researchers, and 35 clinicians. Ninety participants completed Round 2 (43 young people, 14 researchers, and 33 clinicians) and 84 completed Round 3 (41 young people, 11 researchers, and 32 clinicians), indicating strong response rates with low attrition.

Table 1 presents each panel's demographic and relevant characteristics. Most participants were female, with mean age of 18.1 years for the youth panel (SD = 2.2; range 15–23), 42.8 years for the researcher panel (SD = 8.7; range 25–60), and 35.7 years for the clinician panel (SD = 6.4; range 24–52). Nearly all participants were from Australia or Ireland, reflecting the targeted recruitment approach. Over three-quarters of youth participants had accessed digital mental health support more than once (76.9%), and most had accessed services within the past six months (83.8%). Professional panels showed high experience levels: researchers averaged 11.7 years (SD = 6.3) and clinicians averaged 5.3 years (SD = 6.9).

**Table 1:** Characteristics of the three panels.

Characteristic	Youth panel		Researcher panel		Clinician panel	
	*n*	%	*n*	%	*n*	%
Gender						
Men	12	26.1	2	12.5	5	14.3
Women	31	67.4	14	87.5	28	80.0
Gender diverse	3	6.5	-	-	2	5.7
*No response*	*3*					
Country of residence						
Australia	25	64.1	9	64.3	29	85.3
Ireland	14	35.9	1	7.1	5	14.7
Other	-	-	4	28.6	-	-
*No response*	*10*		*2*		*1*	
Access frequency						
Once	9	23.1	n/a		n/a	
More than once	30	76.9	n/a		n/a	
*No response*	*10*					
Access recency						
Within the last 3 months	21	56.8	n/a		n/a	
3–6 months ago	10	27.0	n/a		n/a	
6–12 months ago	3	8.1	n/a		n/a	
More than 12 months ago	3	8.1	n/a		n/a	
*No response*	*12*					
Mean age in years (SD)	18.1 (2.22)		42.8 (8.71)		35.7 (6.43)	
Mean years of experience (SD)	n/a		11.7 (6.33)		5.3 (6.94)	

### 3.2 Client outcomes

Table 2 presents the results for client outcomes. Eleven outcomes reached consensus. All three panels endorsed four outcomes: *Feeling heard and validated*; *Reduced distress; Increased help-seeker capacity;* and *Feeling safe*. Four outcomes were endorsed by youth and researchers, but not clinicians: *Feeling better*; *Reduced hopelessness*; *Increased Coping*; and *Goals, answers, and direction*. Three outcomes were endorsed by clinicians and researchers, but not youth: *Optimism and hope*; *Connection with clinician and service*; and *Reduced overwhelm*.

**Table 2:** Percentage of panelists rating each client outcome as very important or essential in rounds 1 and 2.

Client outcome	Youth panel		Researcher panel		Clinician panel	
	Round 1 (*n* = 49)	Round 2 (*n* = 43)	Round 1 (*n* = 16)	Round 2 (*n* = 14)	Round 1 (*n* = 35)	Round 2 (*n* = 33)
All panel consensus						
Feeling heard and validated	**88**	**91**	**94**	**100**	**97**	**100**
Increased help-seeking capacity	**76**	**88**	**88**	**100**	69	**82**
Reduced distress	73	**81**	**88**	**100**	**83**	**91**
Feeling safe	73	**81**	**93**	**100**	**83**	**88**
Youth/researcher consensus						
Goals, answers and direction	69	**81**	**81**	**86**	*54*	*55*
Feeling better	71	**79**	**88**	**93**	*60*	*67*
Reduced hopelessness	69	**77**	**88**	**93**	*63*	*64*
Increased coping	67	**77**	**81**	**86**	60	67
Clinician/researcher consensus						
Connection with clinician and service	*61*	*72*	**88**	**100**	**77**	**79**
Optimism and hope	*61*	*67*	**88**	**100**	71	**85**
Reduced overwhelm	63	67	**75**	**86**	71	**79**
Single-panel consensus						
Reduced helplessness	67	74	**81**	**93**	66	70
Service experience^a^	–	67	–	**86**	–	70
Relief	59	63	63	**79**	63	67
Clarity and control	59	*63*	69	64	74	**88**
No consensus						
Loneliness	65	74	56	50	43	36
Anxiety	69	70	50	43	60	52
Isolation	59	58	38	21	54	39
Acceptance^a^	–	56	–	43	–	42
Daily functioning	57	56	40	21	43	39
Self-confidence and self-worth	51	53	50	57	54	61
Emotion regulation	49	49	56	50	60	61
Depression	51	49	50	43	46	36
Stress	49	47	50	43	57	61
Mental health literacy	49	42	33	21	63	64
Anger	49	40	63	71	29	18
Community connection	41	40	38	14	29	15
Social and relationships	37	37	33	14	37	33

Results in **bold** (green cells) indicate consensus (≥75% agreement within a panel).

Results in *italics* (orange cells) indicate conflict (≥25% lower agreement than a panel that reached consensus).

a Introduced Round 2.

Only researchers endorsed: *Reduced helplessness*; *Relief;* and *Service Experience*, while only clinicians endorsed *Clarity and control*. Six outcomes were in conflict (see Table 2): *Optimism and hope; Connection with clinician and service; Feeling better; Reduced hopelessness; Goals, answers, and direction;* and *Clarity and control*. Supplementary Table S4 presents the consensus-derived outcomes with descriptions and descriptive statistics (mean rating scores and standard deviations).

### 3.3 Clinician actions

Table 3 presents the results for clinician actions. Fifteen actions reached consensus. All three panels endorsed eight actions: *Manage risk*; *Active listening; Respect diversity*; *Validation; Welcoming environment; Compassion; Checking in;* and *Managing distress*. Two actions were endorsed by youth and researchers, but not clinicians: *Provide resources; Problem-solving*. Four actions were endorsed by clinicians and researchers, but not youth: *Youth-friendly; Set expectations and focus; Give choice; Highlight strengths*. One action was endorsed by youth and clinicians, but not researchers: *Holistic approach.*

**Table 3:** Percentage of panelists rating each clinician action as very important or essential in rounds 2 and 3.

Clinician action	Youth panel		Researcher panel		Clinician panel	
	Round 2 (*n* = 43)	Round 3 (*n* = 41)	Round 2 (*n* = 14)	Round 3 (*n* = 11)	Round 2 (*n* = 33)	Round 3 (*n* = 32)
All panel consensus						
Manage risk	**86**	**93**	**100**	**100**	**100**	**100**
Active listening	**91**	**93**	**86**	**83**	**100**	**100**
Respect diversity	**84**	**90**	**86**	**100**	**91**	**100**
Welcoming environment	**86**	**88**	**93**	**92**	**100**	**100**
Manage distress	**77**	**88**	**93**	**92**	**85**	**94**
Checking in^a^	–	**88**	–	**75**	–	**91**
Validation	**79**	**83**	**100**	**100**	**100**	**100**
Compassion	72	**78**	**93**	**92**	**97**	**100**
Youth/researcher consensus						
Problem solving	**77**	**85**	71	**83**	*39*	*31*
Provide resources	72	**83**	**86**	**100**	*42*	*38*
Clinician/researcher consensus						
Give choice	65	73	**86**	**83**	**76**	**91**
Youth friendly	*56*	*61*	**79**	**83**	**88**	**94**
Set expectations & focus^a^	–	*59*	–	**75**	-	**91**
Highlight strengths	*40*	*41*	64	**83**	**76**	**91**
Youth/clinician consensus						
Holistic approach	74	**78**	*43*	*50*	**85**	**88**
Single-panel consensus						
Action planning	63	68	**86**	**83**	*45*	*34*
Support networks	60	71	*43*	*25*	**79**	**84**
No consensus						
Identify emotions	65	71	36	33	48	50
Skill building	56	66	36	33	58	63
Guided exploration	60	63	29	33	39	25
Goal setting	58	59	50	25	48	34
Helpful before	56	54	36	25	45	38
Reflection	47	44	29	17	33	22

Results in **bold** (green cells) indicate consensus (≥75% agreement within a panel).

Results in *italics* (orange cells) indicate conflict (≥25% lower agreement than a panel that reached consensus).

a Introduced Round 3.

Only clinicians endorsed *Support networks*, while only researchers endorsed *Action planning*. Eight clinician actions were in conflict: *Youth friendly*; *Provide resources*; *Holistic approach*; *Action planning*; *Support networks*; *Highlight strengths*; *Set expectations and focus* and *Problem solving*. Supplementary Table S5 presents the consensus-derived actions with descriptions and descriptive statistics (mean rating scores and standard deviations).

## 4 Discussion

This Delphi study identified and prioritised the client outcomes and clinician actions most important in youth online chat services through consensus among young service users, clinicians, and researchers. A three-round iterative process involving individual ratings and group feedback resulted in 11 client outcomes and 15 clinician actions reaching consensus. This research provides an important step toward developing measurement approaches that address the significant evaluation challenges facing this rapidly expanding service modality.

The 11 consensus-derived client outcomes reflect the core goals achievable for young people within single-session online chat interactions. The agreed outcomes can be organised into five thematic domains: suicidality and self-harm (*Feeling safe*), distress (*Reduced distress, Feeling better*, *Overwhelm),* therapeutic needs (*Feeling heard and validated*, *Connection with clinician and service)*, hope (*Reduced hopelessness, Optimism and hope*), and next steps (*Goals/answers/direction, Increased coping, Increased help-seeking capacity*). Notably, these prioritised outcomes emphasise immediate stabilisation and empowerment, reflecting interest-holders' realistic expectations and understanding of what is possible in single-session online interactions. The importance of feeling safe aligns with recent findings that discussions about suicide are common among youth accessing online chat (53). Interestingly, the low endorsement of outcomes related to social connectedness (*Loneliness, Isolation, Social and relationships, Community connection*), despite their known importance for youth mental health (54), suggests that interest-holders may not view this domain as a feasible priority for online chat services. The SWAN-OM validation study (34) similarly found that outcomes reflecting immediate stabilisation and empowerment were prioritised by young people, while those related to interpersonal functioning were least frequently endorsed.

The 15 consensus-derived clinician actions reflect the core therapeutic processes and behaviours that may contribute to positive outcomes for young people within single-session online chat interactions. These actions can be organised into five thematic domains: responding to distress and risk (*Manage distress, Manage risk*), supportive and respectful engagement (*Welcoming environment*, *Compassion*, *Respect diversity*, *Youth friendly*), active listening (*Active listening*, *Validation*), clarifying immediate needs and goals (*Checking in, Give choice*), and supporting next steps (*Problem solving*, *Provide resources*, *Highlight strengths*). The prioritisation of these actions over more traditional therapeutic techniques (e.g., *Identify emotions*, *Guided exploration*) aligns with existing conceptualisations of online chat as focused on immediate emotional and informational support, rather than longer-term therapeutic change (21). The emphasis on supportive engagement and clarifying immediate needs underscores the distinctive nature of the therapeutic relationship in online chat, where young people maintain greater anonymity and control than in traditional settings (24).

The findings reveal meaningful differences in how researchers, clinicians, and young people prioritise client outcomes in online chat settings. Clinicians were more likely to endorse *Optimism and hope*, while young people more strongly endorsed *Reduced hopelessness*, suggesting that young help-seekers focus primarily on alleviating negative mental states rather than building positive ones – a pattern consistent with research in crisis helpline contexts (42). The divergences between clinicians and young people extend to therapeutic focus: clinicians' stronger endorsement of *Connection with clinician and service* likely reflects their professional emphasis on relationship-building as foundational to effective support, while young people's preference for *Coping* and *Goals, answers, and direction* suggests a more instrumental, solutions-focused need, consistent with recent findings from a UK text-based support service (55).

Divergent views on clinician actions across panels provide further insights into interest-holder perspectives. Young people's stronger endorsement of *Provide resources* and *Problem-solving* reflects their desire for immediate, tangible support, whereas clinicians prioritised therapeutic techniques such as *Highlight strengths* and *Holistic approach*. This suggests young people may view clinicians as facilitators who connect them to external resources and provide guidance on next steps, while clinicians prioritise understanding, learning, and strategies delivered within the chat session. However, clinicians' lower endorsement of practical outcomes and actions may also reflect professional caution about being overly directive, rather than dismissing their importance. Clinicians' higher endorsement of *Youth-friendly*, *Set expectations and focus,* and *Give choice* likely reflects their professional understanding of service parameters and intervention frameworks that young people may not be cognisant of. These varied perspectives reveal important opportunities for improving service alignment through enhanced shared decision-making processes and clearer communication about service goals, while highlighting the need for comprehensive measurement approaches that capture a broad range of outcomes and actions.

### 4.1 Implications for research and practice

The client outcomes and clinician actions identified in this Delphi study can advance research, practice, and policy in youth mental health services. These consensus-based priorities provide a foundation for developing measurement tools specifically tailored to single-session online chat services, enabling rigorous and consistent evaluation. The breadth of items supports the growing movement toward idiographic measurement approaches that reflect young people's individual goals and needs, consistent with person-centred care principles (34, 41, 56). Identifying key clinician actions addresses a critical gap in understanding therapeutic processes in online chat contexts (29), providing direction for process evaluation and understanding mechanisms of change (31). Our findings also contribute to the broader youth single-session therapy literature by identifying outcomes and actions specific to youth online chat contexts. Comparing the priorities identified here with those in other SST research could inform service design and training and enhance alignment between digital and face-to-face approaches (34, 57, 58).

Services can use these findings to refine their frameworks and training programs, particularly regarding the balance between practical solutions and therapeutic engagement. Researchers can use them to inform programme theory development for youth online chat interventions, promoting shared understanding and transferability across settings (35). Future research should examine how specific clinician actions relate to client outcomes, as this underdeveloped area is critical for understanding mechanisms of change in youth online chat support and other single-session interventions (30, 34). It should also explore how panel differences influence service delivery and outcomes, for example, whether clinicians' emphasis on relational processes vs. young people's preference for practical solutions affects engagement, satisfaction, or therapeutic change.

While this study establishes consensus on what to measure, implementation will require careful attention to feasibility constraints, data collection methods, and alignment with service models. Challenges remain in applying traditional pre-post self- or clinician-report measures in brief online chat contexts. Innovative approaches, such as machine learning techniques for automated evaluation, hold promise for overcoming these barriers (33, 34), although ethical and practical challenges require careful consideration.

Finally, young people's emphasis on immediate relief, practical next steps, and help-seeking capacity supports policy frameworks that position online chat services as critical entry points to mental health systems, with implications for service integration (17, 18). Young people clearly want direction on what to do next, which may seem at odds with less directive, client-centred approaches traditionally favoured by clinicians, despite some research showing the benefits of more directive approaches (59). Navigating this tension between providing tangible next steps while preserving autonomy and validating feelings and experiences, will be essential for online clinicians and service designers.

### 4.2 Strengths and limitations

A key strength of this study was the three-panel online Delphi methodology, which enabled us to integrate three distinct and valuable perspectives, while maintaining participant anonymity and minimising power imbalances. The high response rate showed strong participant engagement throughout the Delphi process and supports the validity of our findings (40). This is the first consensus study to establish measurement priorities specifically for single-session online chat support for young people's mental health.

Several limitations affect the generalisability of these findings. We used purposive sampling from two national youth mental health services for youth and clinician participants, and all participants self-selected to join the study. This recruitment approach may have introduced selection bias, as panelists were likely more engaged with the topic than the broader population of service users and clinicians.

Sample composition presents additional limitations. The researcher panel was smaller than the target sample size of 20, with some dropout, potentially limiting the representativeness of this interest-holder perspective in the consensus-building process. Most panelists were from Australia or Ireland, constraining the extent to which findings can be considered internationally representative. Caution is therefore needed when generalising findings to other youth online chat services. Priority outcomes and actions may differ in services operating under different delivery models, language and cultural contexts, and resource environments, particularly in low- and middle-income countries. The youth panel age range (15–25 years) also excludes early adolescents (12–14 years) typically served by these platforms. Further research should examine whether the identified outcomes and actions are similarly prioritised across other geographic and cultural contexts and among early adolescents.

## 5 Conclusion

Youth online chat services represent a critical and rapidly expanding approach to mental health service delivery that is well-positioned to provide accessible and timely support at scale. However, the unique characteristics of single-session online chat interactions have created significant challenges for outcome measurement and service evaluation, with traditional approaches proving inadequate. This Delphi study takes an important step toward overcoming these challenges by establishing a consensus-based framework of priority client outcomes and clinician actions specifically tailored to single-session online chat support for young people. These findings provide a valuable foundation for developing novel measurement approaches that enable services to monitor effectiveness and optimise online chat interventions for young people.

## Data Availability

The raw data supporting the conclusions of this article will be made available by the authors, without undue reservation.
