# Supplementary material for: Consensus derived client outcomes and clinician actions for youth online chat mental health services: a Delphi study
Source: Front Digit Health. 2025 Dec 16;7:1671364. doi: 10.3389/fdgth.2025.1671364 (PMC12748242; doi:10.3389/fdgth.2025.1671364)
Supplement: Supplementary file 2 [file Table1.pdf]

Supplementary Material

Table S1: Item Mapping and Sources: Client Outcomes

Label for Survey	Description for Survey	Source Item	Reference
**Distress**	Young person's emotional distress has decreased	Distress	COS (Curll et al., 2024)
**Feeling better**	Young person feels better	Feel better	SWAN-OM (De Ossorno Garcia et al., 2021)
**Optimism and hope**	Young person feels more optimistic or hopeful about their way forward (i.e., they see that things in their life could improve)	Hope	Systematic review (Tibbs et al., 2025)
		Increased optimism and hope for the future	eheadspace evaluation framework (hNYMHF, 2024)
		Demonstrate ambition and hope for the future	Kooth ToC (Hanley et al., 2021)
		Able to consider future strategies	Kooth ToC (Hanley et al., 2021)
**Self-confidence and worth**	Young person feels better about themselves. Young person is more aware of their strengths and value as a person	Improved feelings of self-confidence and worth	eheadspace evaluation framework (hNYMHF, 2024)
		Increased confidence, personal responsibility and ability to make decisions	Kooth ToC (Hanley et al., 2021)
**Relief**	Young person experiences a sense of catharsis or relief	Cartharsis/Relief	Programme Theory Model of Jigsaw Live Chat (Tibbs, 2024)
**Feeling heard**	Young person feels heard, validated, not judged and understood	Feeling Heard	COS (Curll et al., 2024)
		Feel listened to	SWAN-OM (De Ossorno Garcia et al., 2021)
		Feeling listened to, understood and supported	eheadspace evaluation framework (hNYMHF, 2024)
		YP feels validated and understood in their experience	Programme Theory Model of Jigsaw Live Chat (Tibbs, 2024)
		Feels heard and has feelings validated	Kooth ToC (Hanley et al., 2021)

**Community connection**	Young person feels part of a community and sense of belonging	Increased sense of belonging and connection to community and culture	eheadspace evaluation framework (hNYMHF, 2024)
		Feels a sense of community	Kooth ToC (Hanley et al., 2021)
		Builds connections and a safe online and offline community	Kooth ToC (Hanley et al., 2021)
**Connection with clinician/service**	Young person feels rapport with and trust in service provider and service	Connection with clinician/service	COS (Curll et al., 2024)
		Rapport with and trust in providers and services	eheadspace evaluation framework (hNYMHF, 2024)
		Therapeutic alliance developed with clinician	Programme Theory Model of Jigsaw Live Chat (Tibbs, 2024)
		Has experienced opening up to someone and built a relationship with a professional	Kooth ToC (Hanley et al., 2021)
**Coping**	Young person feels more confident and motivated to manage or cope with their situation	Find ways i can help myself	SWAN-OM (De Ossorno Garcia et al., 2021)
		Learn how to feel better	SWAN-OM (De Ossorno Garcia et al., 2021)
		Identify ways to help me worry less	SWAN-OM (De Ossorno Garcia et al., 2021)
		Increased knowledge, skills and strategies to manage mental health issues and challenges	eheadspace evaluation framework (hNYMHF, 2024)
		Increased capacity to self-monitor and manage mental health and wellbeing	eheadspace evaluation framework (hNYMHF, 2024)
		YP understands their resources and coping strategies	Programme Theory Model of Jigsaw Live Chat (Tibbs, 2024)
		Better able to manage current and future situations	Kooth ToC (Hanley et al., 2021)
**Increased help-seeking capacity**	Increased capacity, willingness, and confidence to seek support for mental health issues and needs in future (e.g. from family and friends, or from services)	Increased confidence and capacity to seek mental health and wellbeing support in future	eheadspace evaluation framework (hNYMHF, 2024)
		Increased feelings of safety engaging with support	eheadspace evaluation framework (hNYMHF, 2024)
		Be comfortable asking for help outside Kooth	SWAN-OM (De Ossorno Garcia et al., 2021)

		Find out how helpful it is to talk to someone	SWAN-OM (De Ossorno Garcia et al., 2021)
		Be able to open up to people in my life	SWAN-OM (De Ossorno Garcia et al., 2021)
		Reinforcement of helpseeking	Programme Theory Model of Jigsaw Live Chat (Tibbs, 2024)
		Is aware that ongoing support is available - is not alone	Kooth ToC (Hanley et al., 2021)
		Starts to engage or has information about face-to-face services	Kooth ToC (Hanley et al., 2021)
**Goals, answers & direction**	Young person has identified positive next steps. This can include moving toward more adaptive coping strategies.	Next Steps	COS (Curll et al., 2024)
		Learn the steps to achieve something I want	SWAN-OM (De Ossorno Garcia et al., 2021)
		Increased capacity to set and work towards goals	eheadspace evaluation framework (hNYMHF, 2024)
		Achieves personal goals and recognises progress made	Kooth ToC (Hanley et al., 2021)
		Sets personal goals for change	Kooth ToC (Hanley et al., 2021)
		Changes perspective or sees new options	Kooth ToC (Hanley et al., 2021)
**Sense of clarity and control over their situation**	Young person feels a sense of choice, influence, and control over how they navigate their situation (e.g., recognising where support is available)	Identify a solution to a problem in my life	SWAN-OM (De Ossorno Garcia et al., 2021)
		Sense of clarity and control over their situation	eheadspace evaluation framework (hNYMHF, 2024)
		Coping/helpseeking efficacy	Programme Theory Model of Jigsaw Live Chat (Tibbs, 2024)
		Takes ownership of an issue	Kooth ToC (Hanley et al., 2021)
**Helplessness**	Young person feels less powerless to improve their situation	Helplessness	COS (Curll et al., 2024)
**Overwhelm**	Young person feels less overwhelmed	Overwhelm	COS (Curll et al., 2024)
**Isolation**	Young person feels less isolated	Reduced sense of isolation	eheadspace evaluation framework (hNYMHF, 2024)
**Loneliness**	Young person feels less lonely	Feeling less lonely	eheadspace evaluation framework (hNYMHF, 2024)

**Depression**	Young person feels less depressed and improved mood	Depression	Systematic review (Tibbs et al., 2025)
		Psychological distress	Systematic review (Tibbs et al., 2025)
		Reduced symptoms of psychological distress	eheadspace evaluation framework (hNYMHF, 2024)
**Anxiety**	Young person feels less anxious, worried, fearful, panicked	Anxiety	Systematic review (Tibbs et al., 2025)
		Psychological distress	Systematic review (Tibbs et al., 2025)
		Reduced symptoms of psychological distress	eheadspace evaluation framework (hNYMHF, 2024)
**Stress**	Young person feels less stressed	Acknowledges a reduction in stress	Kooth ToC (Hanley et al., 2021)
		Psychological distress	Systematic review (Tibbs et al., 2025)
		Reduced symptoms of psychological distress	eheadspace evaluation framework (hNYMHF, 2024)
**Hopelessness**	Young person feels less hopeless	Hopelessness	COS (Curll et al., 2024)
**Mental health literacy**	Young person has a better awareness and understanding of their mental health	Increased awareness and understanding of current mental health issues and situation	eheadspace evaluation framework (hNYMHF, 2024)
		Increased mental health literacy	eheadspace evaluation framework (hNYMHF, 2024)
		Improved attitude/reduced stigma towards mental health issues and seeking support	eheadspace evaluation framework (hNYMHF, 2024)
		Understand my feelings and/or behaviours	SWAN-OM (De Ossorno Garcia et al., 2021)
		Better understanding of mental health difficulties	Programme Theory Model of Jigsaw Live Chat (Tibbs, 2024)
		Able to reflect on thoughts, feelings, and perceptions	Kooth ToC (Hanley et al., 2021)
		Gets information	Kooth ToC (Hanley et al., 2021)
**Feeling safe**	Young person feels safer in their environment or relationships (IPV, crime). Reduced risk of intentionally hurting themselves (suicide, self-harm)	NSSI Risk	COS (Curll et al., 2024)
		Suicide Risk	COS (Curll et al., 2024)
		Feel safe in my relationships	SWAN-OM (De Ossorno Garcia et al., 2021)
		Find information about how to keep myself safe	SWAN-OM (De Ossorno Garcia et al., 2021)
		Safer or crisis reduced	Kooth ToC (Hanley et al., 2021)

**Social and relationships**	Improved communication skills with others in their life. Skills in managing conflict. Getting along better with family and friends	Learn how to relate to other people	SWAN-OM (De Ossorno Garcia et al., 2021)
		Learn how to manage conflict with others	SWAN-OM (De Ossorno Garcia et al., 2021)
		Identify solutions to improve my relationships	SWAN-OM (De Ossorno Garcia et al., 2021)
		Improved relationships with family and friends	eheadspace evaluation framework (hNYMHF, 2024)
**Daily functioning**	Young person feels able to perform their daily activities including self-care, work/school. Improved functioning (e.g., sleep)	Increased capacity to manage daily life and activities	eheadspace evaluation framework (hNYMHF, 2024)
**Emotion regulation**	Young person feels better able to deal with/regulate their emotions	Be more comfortable with my feelings	SWAN-OM (De Ossorno Garcia et al., 2021)
		Increased self-awareness of emotions and behaviours, and capacity to apply knowledge, skills and strategies in future	eheadspace evaluation framework (hNYMHF, 2024)
		Greater self-awareness and emotion regulation	Kooth ToC (Hanley et al., 2021)
**Anger**	Young person feels less angry or frustrated		Recommendation from research team

Table S2: Item Mapping and Sources: Clinician Actions

Label for Survey	Description for Survey	Source Item	Reference
**Welcoming environment**	Foster a warm, safe, and non-judgmental space for open sharing.	Creating a warm, safe, and empathic space	Programme Theory Model of Jigsaw Live Chat (Tibbs, 2024)
**Validation**	Acknowledge and normalize young person's feelings, reassuring them that their emotions are valid	Validation and normalising of experiences	Programme Theory Model of Jigsaw Live Chat (Tibbs, 2024)
		Not letting the help-seeker feel like their issue is not important/invalid	eheadspace evaluation framework (hNYMHF, 2024)
**Active listening**	Engage attentively and supportively with young person to show understanding.	Active listening	Programme Theory Model of Jigsaw Live Chat (Tibbs, 2024)
		Listen to the young person, giving them space to share, and asking for their feedback about services provided or discussed.	eheadspace evaluation framework (hNYMHF, 2024)
**Compassion**	Showing a strong sense of empathy and compassion	Building an empathic relationship	Kooth ToC (Hanley et al., 2021)
		Compassionate; Having a strong sense of empathy and understanding	eheadspace evaluation framework (hNYMHF, 2024)
**Action planning**	Identification of next steps	Action planning	Systematic review (Tibbs et al., 2025)
		Identification of next steps	Programme Theory Model of Jigsaw Live Chat (Tibbs, 2024)
**Highlight strengths**	Acknowledge the inherent skills and resources that young people already have	Acknowledge strengths	Programme Theory Model of Jigsaw Live Chat (Tibbs, 2024)
**Manage distress**	Evaluate distress levels and tailor appropriate responses	Recognising and acknowledging **level of distress**, potential risk, and reasons for attendance	Programme Theory Model of Jigsaw Live Chat (Tibbs, 2024)

		Assessing **distress** and risk and tailoring responses	Kooth ToC (Hanley et al., 2021)
**Manage risk**	Evaluate suicidality and risk of harm. Undertake safety planning	Recognising and aknowledging level of distress, **potential risk**, and reasons for attendance	Programme Theory Model of Jigsaw Live Chat (Tibbs, 2024)
		Assessing distress and **risk** and tailoring responses	Kooth ToC (Hanley et al., 2021)
**What's been helpful before**	Identify past successes and strategies that have been effective	Focus on past successes	Systematic review (Tibbs et al., 2025)
		Identifying what has helped before	Kooth ToC (Hanley et al., 2021)
		Acknowledging the inherent skills and resources that young people already have	eheadspace evaluation framework (hNYMHF, 2024)
**Personal reflection**	Encourage self-reflection and provide insight	Encouraging **reflection** and taking responsibility	Kooth ToC (Hanley et al., 2021)
		Be needs-led & responsive	eheadspace evaluation framework (hNYMHF, 2024)
**Give choice**	Empower the young person to make decisions, promote autonomy (independence) and support those decisions	Encouraging reflection and **taking responsibility**	Kooth ToC (Hanley et al., 2021)
		Give the choice to young person to make decisions (promote autonomy) and support those decisions	eheadspace evaluation framework (hNYMHF, 2024)
**Youth friendly**	Use age-appropriate language and concepts	Provide youth-friendly & age-appropriate services	eheadspace evaluation framework (hNYMHF, 2024)
**Respect diversity**	Be culturally aware and sensitive, ensure care is respectful and relevant to diverse backgrounds and identities	Culturally aware and safe - understanding of our cultural roles and expectations and experiences	eheadspace evaluation framework (hNYMHF, 2024)

**Problem-solving**	Help analyse the young person's situation and explore strategies to resolve problems	Problem-solving	Systematic review (Tibbs et al., 2025)
		Cocreating goals and **solutions** with young people	Kooth ToC (Hanley et al., 2021)
**Goal setting**	Agree on a goal to work towards a positive outcome for the young person	Goal setting	Systematic review (Tibbs et al., 2025)
		Cocreating **goals** and solutions with young people	Kooth ToC (Hanley et al., 2021)
		Follow-up plans, or next steps will be discussed so the young person is clear about what they need to do next	eheadspace evaluation framework (hNYMHF, 2024)
**Support networks**	Explore and develop both formal and informal support networks	Exploration and identification of formal and informal supports	Programme Theory Model of Jigsaw Live Chat
		Exploring the young person’s relationship and support systems	Kooth ToC (Hanley et al., 2021)
**Provide resources**	Provide clear and informative resources for the young person regarding their mental health and avenues of support	Giving information and signposting	Kooth ToC (Hanley et al., 2021)
**Guided exploration**	Help young people understand their situations by breaking it down into smaller parts and exploring different perspectives	Youth-led problem exploration	Programme Theory Model of Jigsaw Live Chat
**Skill building**	Provide psychoeducation and skills training to empower clients in managing their mental health proactively	Instructions on how to perform behaviour	Systematic review (Tibbs et al., 2025)
**Holistic approach**	Focus on the individual as a whole person rather than just their problems, considering their physical, emotional, and social well-being.	Seeing the client as a person, not just their problems	eheadspace evaluation framework (hNYMHF, 2024)
**Help identify emotions**	Help the young person to better understand and express their emotions		Recommendation from research team

Table S3: Details of items that were modified or added between rounds.

**Label**	**Description**
Client Outcomes (Rounds 1-2)	
Relief	Young person experiences a sense of catharsis or relief **(e.g. like a weight has been lifted off their shoulders)**
Feeling heard **& validated**	Young person feels heard**, validated, not judged** and understood
Increased help-seeking capacity	Increased capacity, willingness, and confidence to seek support for mental health issues and needs in future **(e.g. from family and friends, or from services)**
Social and relationships	Improved communication skills with others in their life. Skills in **assertiveness and** managing conflict. Getting along better with family and friends
Daily functioning	Young person feels able to perform their daily activities including self-care, work/school. Improved functioning (e.g., sleep, **routines**)
**[NEW] Acceptance**	**Young person is more able to accept themselves (e.g. identity) and/or their situation (e.g. current stressors)**
**[NEW] Service experience**	**Reflects a young person's positive or negative overall experience of the service**
Clinician Actions (Rounds 2-3)	
**[NEW] Checking in**	**Check in with the young person to ensure the chat is on track for them (e.g. Is it ok to talk about this? Am I getting this right?)**
**[NEW] Set expectations & focus**	**Set expectations of what the service can provide and agree on the focus to ensure time is used effectively**

Note. Text in bold reflects additional or modified item label or description.

Table S4: Consensus derived outcomes: Descriptions and descriptive statistics.

Outcome	Description	Overall[a]		Youth		Researcher		Clinician	
		GM	Rank	*M*	*SD*	*M*	*SD*	*M*	*SD*
Feeling heard and validated	Young person feels heard, validated, not judged and understood	4.88	1	4.70	0.64	5.00	0.00	4.94	0.24
Feeling safe	Young person feels safer in their environment or relationships (IPV, crime). Reduced risk of intentionally hurting themselves (suicide, self-harm)	4.46	2	4.19	1.26	4.79	0.43	4.39	0.70
Reduced distress	Young person's emotional distress has decreased	4.39	3	4.26	0.82	4.71	0.47	4.21	0.60
Connection with clinician and service	Young person feels rapport with and trust in service provider and service	4.27	4	4.16	1.02	4.21	0.43	4.45	0.83
Increased help-seeking capacity	Increased capacity, willingness, and confidence to seek support for mental health issues and needs in future (e.g. from family and friends, or from services)	4.27	5	4.23	0.72	4.43	0.51	4.15	0.71
Optimism and hope	Young person feels more optimistic or hopeful about their way forward (i.e., they see that things in their life could improve)	4.18	6	4.05	0.95	4.50	0.52	4.00	0.56
Reduced hopelessness	Young person feels less hopeless	4.11	7	4.19	0.85	4.50	0.65	3.64	0.74
Goals, answers and direction	Young person has identified positive next steps. This can include moving toward more adaptive coping strategies	4.07	8	4.28	0.88	4.21	0.89	3.73	0.76
Reduced overwhelm	Young person feels less overwhelmed	4.02	9	4.05	0.84	4.14	0.66	3.88	0.55
Feeling better	Young person feels better	3.97	10	4.16	0.95	3.93	0.62	3.82	0.68
Increased coping	Young person feels more confident and motivated to manage or cope with their situation	3.96	11	4.09	0.75	4.00	0.55	3.79	0.65

[a]Based on the grand mean (mean of means).

Table S5: Consensus derived clinician actions: Descriptions and descriptive statistics.

Outcome	Description	Overall [a]		Youth		Researcher		Clinician	
		GM	Rank	*M*	*SD*	*M*	*SD*	*M*	*SD*
Manage Risk	Evaluate suicidality and risk of harm. Undertake safety planning	4.77	1	4.61	0.80	4.83	0.39	4.88	0.34
Active Listening	Engage attentively and supportively with young person to show understanding	4.71	2	4.61	0.63	4.58	0.79	4.94	0.25
Respect Diversity	Be culturally aware and sensitive, ensure care is respectful and relevant to diverse backgrounds and identities	4.71	3	4.46	0.84	4.75	0.45	4.91	0.30
Validation	Acknowledge and normalize young person's feelings, reassuring them that their emotions are valid	4.68	4	4.29	1.01	4.83	0.39	4.91	0.30
Welcoming Environment	Foster a warm, safe, and non-judgmental space for open sharing	4.66	5	4.41	0.92	4.67	0.65	4.91	0.30
Compassion	Showing a strong sense of empathy and compassion	4.47	6	4.10	0.86	4.50	0.67	4.81	0.40
Checking In	Check in with the young person to ensure the chat is on track for them (e.g. Is it ok to talk about this? Am I getting this right?)	4.36	7	4.32	0.76	4.42	1.08	4.34	0.65
Manage Distress	Evaluate distress levels and tailor appropriate responses	4.33	8	4.22	0.65	4.25	0.62	4.53	0.62
Youth Friendly	Use age-appropriate language and concepts	4.29	9	3.85	1.26	4.42	0.79	4.59	0.71
Holistic Approach	Focus on the individual as a whole person rather than just their problems, considering their physical, emotional, and social well-being	4.14	10	4.20	0.78	3.50	1.38	4.72	0.68
Set Expectations & Focus	Set expectations of what the service can provide and agree on the focus to ensure time is used effectively	4.09	11	3.88	0.90	4.08	1.00	4.31	0.86
Give Choice	Empower the young person to make decisions, promote autonomy (independence) and support those decisions	4.01	12	3.76	0.99	4.08	0.67	4.19	0.59
Provide Resources	Provide clear and informative resources for the young person regarding their mental health and avenues of support	3.99	13	4.07	0.72	4.58	0.51	3.31	0.69
Highlight Strengths	Acknowledge the inherent skills and resources that young people already have	3.86	14	3.54	0.84	3.92	0.51	4.13	0.55
Problem Solving	Help analyse the young person's situation and explore strategies to resolve problems	3.81	15	4.15	0.65	4.00	0.60	3.28	0.73

[a]Based on the grand mean (mean of means).

References

Curll S, Mazzer K, Rickwood D. The development of a core outcome set for crisis helplines: a three-panel Delphi study. *J Affect Disord Rep*. (2024) 16:100763. doi: 10.1016/j.jadr.2024.100763

De Ossorno Garcia S, Salhi L, Sefi A, Hanley T. The session wants and needs outcome measure: the development of a brief outcome measure for single-sessions of web-based support. *Front Psychol*. (2021) 12:748145. doi: 10.3389/fpsyg.2021.748145

Hanley T, Sefi A, Grauberg J, Prescott J, Etchebarne A. A theory of change for web-based therapy and support services for children and young people: collaborative qualitative exploration. *JMIR Pediatr Parent*. (2021) 4:1. doi: 10.2196/23193

headspace National Youth Mental Health Foundation (hNYMHF). eheadspace evaluation framework summary. (2024). https://headspace.org.au/assets/Reports/eheadspace-evaluation-framework-summary.pdf

Tibbs M, O'Reilly MD, Carey A, Moore J, Fitzgerald A. Co-creation of a program theory and evaluability assessment of an Irish live chat intervention for youth mental health. *Front Digit Health*. (in press).

Tibbs M. Online communication and youth mental health (Unpublished doctoral thesis). University College Dublin, School of Psychology. (2025). https://researchrepository.ucd.ie/entities/publication/3708b33e-dddf-4987-9deb-4c2d986c0ce6
